# LC**–**MS/MS-based multiplex antibacterial platform for therapeutic drug monitoring in intensive care unit patients

**DOI:** 10.3389/fphar.2023.1116071

**Published:** 2023-04-18

**Authors:** Liang Liu, Liu Zhang, Xiangyi Zheng, Xing Liu, Wei Liu, Jianhua Wu

**Affiliations:** ^1^ Department of Pharmacy, Zhongnan Hospital of Wuhan University, Wuhan, China; ^2^ Department of Critical Care Medicine, Zhongnan Hospital of Wuhan University, Wuhan, China; ^3^ School of Physics and Technology, Wuhan University, Wuhan, China

**Keywords:** antibacterial agents, multiplex analysis, therapeutic drug monitoring, LC-MS/MS, MRM, ICU

## Abstract

Empirically prescribed standard dosing regimens of antibacterial agents may result in insufficient or excess plasma concentrations with persistently poor clinical outcomes, especially for patients in intensive care units (ICUs). Therapeutic drug monitoring (TDM) of antibacterial agents can guide dose adjustments to benefit patients. In this study, we developed a robust, sensitive, and simple liquid chromatography-tandem mass spectrometry (LC–MS/MS) platform for the quantification of 14 antibacterial and antifungal agents (beta-lactams piperacillin, cefoperazone, and meropenem; beta-lactamase inhibitors tazobactam and sulbactam; antifungal agents fluconazole, caspofungin, posaconazole, and voriconazole; and daptomycin, vancomycin, teicoplanin, linezolid, and tigecycline) that can be used for patients with severe infection. This assay requires only 100 µL of serum with rapid protein precipitation. Chromatographic analysis was performed using a Waters Acquity UPLC C8 column. Three stable isotope-labeled antibacterial agents and one analogue were used as internal standards. Calibration curves ranged from 0.1–100 μg/mL, 0.1–50 μg/mL, and 0.3–100 μg/mL for different drugs, and all correlation coefficients were greater than 0.9085. Intra- and inter-day imprecision and inaccuracy values were below 15%. After validation, this new method was successfully employed for TDM in routine practice.

## 1 Introduction

Antibacterial therapy is essential for the management of infections in intensive care unit (ICU) patients. (Roberts et al., 2014). However, the current empirically prescribed standard dosing regimens consider only the patients body weight and neglect other factors, often resulting in insufficient or excess plasma concentrations that persistently have poor clinical outcomes. (Garnacho-Montero et al., 2003; Zaragoza et al., 2003). Furthermore, the inappropriate use of antibacterial agents can shorten the clinical lifespan of these currently available drugs because of the increasing antibacterial resistance worldwide. Critically ill patients experience extensive physiological alterations in the form of liver or kidney failure, extravascular loss of fluids, inflammation associated with sepsis, and shock. (Hosein et al., 2011; Pea et al., 2018). Such events can modify the pharmacokinetics of antibacterial agents, yielding heterogeneous pharmacokinetic (PK) parameters. Furthermore, medical procedures such as extra-renal purification, mechanical ventilation, vascular replacement, or extracorporeal circulation can influence the PK parameters of antibacterial agents. (Asín-Prieto et al., 2015; Maguigan et al., 2021). Because of the unstable condition of ICU patients, the distribution and elimination of antibacterial agents in these patients are inconsistent, causing large variations in serum levels even if the recommended dosing regimen is followed. Mounting evidence has shown the benefits of achieving PK/pharmacodynamic (PK/PD) goals, which include reduced mortality, toxicities, burden, and length of hospital stays. (Revilla et al., 2007; Scaglione and Paraboni, 2008; Adembri et al., 2020; Coste et al., 2020). This highlights the need for developing advanced approaches to implement PK/PD strategies in clinical practice.

With increasing understanding of the associations among antibacterial agent dosing, PK/PD exposure and clinical outcomes in patients, a strong perception of personalized antibacterial agent dosing in critically ill patients has emerged with the help of therapeutic drug monitoring (TDM). (Bengtsson, 2004; Ashbee et al., 2014; Parker et al., 2015; Muller et al., 2018; Garzón et al., 2020; Mabilat et al., 2020). Various methods have been employed to quantify antibacterial agents in serum levels. The most commonly used methods are antibody-based immunoassays, which include the electrochemiluminescence immunoassay, (Vogeser et al., 2014), enzyme multiplied immunoassay, (Clarke, 2004), and enzyme-linked immunosorbent assay. (Uglietti et al., 2007; Wu et al., 2012). Although these techniques can be readily used in laboratories, the data obtained using these techniques can be erroneous as they are easily altered by the presence of human antibodies in specimens, which may bind to various components within the assay system. Numerous high-performance liquid chromatography-ultraviolet (HPLC-UV) methods have been reported for the determination of antibacterial agents in serum samples. However, these methods require a large sample volume and lack multiplexing ability. (Malfará et al., 2007; Franco et al., 2016). Liquid chromatography-tandem mass spectrometry (LC–MS/MS) is an alternative analytical technique used in the field of quantitative bioanalysis, including TDM. (Müller and Rentsch, 2010; Adaway and Keevil, 2012). This technique provides a combination of high sensitivity, high throughput, wide dynamic range, multiplexing ability, and good reproducibility, and requires low sample volumes. These features are particularly suitable for the simultaneous quantification of multiple antibacterial agents in a single biological sample. Several reported LC–MS/MS-based assays can simultaneously quantify six or more antibacterial agents in human plasma or serum samples. (Ohmori et al., 2011; Oswald et al., 2011; Ferrari et al., 2019; Barco et al., 2020; Rehm and Rentsch, 2020; Feliu et al., 2021). However, considering the high risk of fungal infections in ICUs, antifungal drugs must be involved.

This study describes a new LC–MS/MS platform for the sensitive and quantitative analysis of 14 antibacterial and antifungal agents for routine TDM. These agents include piperacillin, tazobactam, cefoperazone, sulbactam, meropenem, daptomycin, vancomycin, teicoplanin, linezolid, fluconazole, caspofungin, posaconazole, voriconazole, and tigecycline. These agents are commonly used for treating severe infections or drug-resistant infections in ICUs, and the benefits of the selected antibacterial agents to achieve PK/PD targets have been reported previously. (Asín-Prieto et al., 2015).

## 2 Materials and methods

### 2.1 Chemicals and reagents

In this study, certified reference materials were used. Linezolid (LIN, purity 98%), caspofungin (CAS, purity >90%), and teicoplanin (TEI, purity 98%) were purchased from TRC (Toronto, Canada). Daptomycin (DAP, purity 95%) and the internal standard (IS) daptomycin-d5 trifluoroacetic (DAP-d5, purity 95%) were obtained from Hengrui Pharmaceutical Co., Ltd. (Jiangsu, China). Piperacillin (PIP, purity 95.2%), [^2^H_5_]-piperacillin sodium salt (PIP-IS, purity 95.2%), cefoperazone (CEF, purity 93.09%), and tazobactam (TAZ, purity 98%) were obtained from LGC Standards Ltd. (Molsheim, France). Ethylparaben (ETH, purity 99%, used as IS) was procured from Sigma Aldrich (Missouri, United States). Meropenem (MER, purity 98%) was purchased from Sumitomo Dainippon Pharma Co., Ltd. (Osaka, Japan). Fluconazole (FLU, purity 99.8%) and sulbactam (SUL, purity 99.5%) were purchased from ANPEL-TRACE Standard Technical Services Co. Ltd. (Shanghai, China). Vancomycin (VAN, purity 98%) was purchased from Vianex S.A. (Plant C) (Athens, Greece). Tigecycline (TIG, purity 96%) was purchased from Hisun Pharm Co. Ltd. (Zhejiang, China). Voriconazole (VOR, purity >99.5%) and [^13^C_2_,^2^H_3_]- voriconazole (VOR-IS, purity >99.5%) were purchased from Sichuan Meidakang Huakang Pharmaceutical Co. Ltd. (Sichuan, China). Posaconazole (POS, purity 99.9%) was obtained from CATO (Eugene, Oregon, United States). HPLC-grade acetonitrile (ACN), methanol (MeOH), and formic acid (FA) were purchased from Thermo Fisher Scientific (Waltham, MA, United States). Ultrapure water was generated using a Clever-S ultrapure water machine (Shanghai, China). Chromatography-grade methanol and acetonitrile were obtained from Tedia company (Ohio, United States). Dimethyl sulfoxide (DMSO) was purchased from Meryer Co., Ltd. (Shanghai, China).

### 2.2 Collection of serum

Blood samples were obtained from healthy volunteers and ICU patients at Zhongnan Hospital of Wuhan University under the guidance of the ethical review committee [2022238K]. All participants signed an informed consent form. Serum samples were collected in a yellow separate glue coagulant tube and centrifuged at 4500 rpm (1940g) for 10 min; the supernatant was stored at −80°C until analysis.

### 2.3 Sample preparation

The serum samples were thawed at room temperature, and 100 μL of these samples was added to a polypropylene tube. Next, 20 μL of 40 μg/mL mixed IS solution was added to this solution, following 480 μL of methanol (0.1% FA) was added to induce precipitation. After vortexing for 1 min, the samples were centrifuged at 12,000 rpm (13780 g) for 8 min. Finally, 500 μL of the supernatant was transferred to a vial for analysis.

### 2.4 Chromatographic and mass spectrometric conditions

LC–MS/MS analyses were performed using an LCMS-8050 triple-quadrupole mass spectrometer (Shimadzu, Japan). Chromatographic analyses were conducted on a Waters Acquity UPLC C_8_ column (1.7 μm, 2.1 mm × 50 mm). The column temperature was maintained at 40°C and 2 μL of the sample was injected into the LC-30A UPLC system. Mobile phase A was 0.1% FA in water, and mobile phase B was 0.1% FA in acetonitrile. The flow rate was set to 0.4 mL/min. The gradient was set as follows: B, 5% (0 min) **→** 5% (0.5 min) **→** 50% (3 min) **→** 100% (4 min) **→** 100% (7 min) **→** 5% (9 min) **→** 5% (10 min). The total run time was 10 min. The gradient was shown in Supplementary Table S1.

The following interface settings were employed for sample analysis: ion transfer tube temperature, 300°C; sheath gas flow, 3.0 L/min; auxiliary gas flow, 10.0 L/min; and vaporizer temperature, 400°C. The desolvation temperature was maintained at 250°C. Quantification was performed by multiple reaction monitoring (MRM).

### 2.5 Stock solutions, calibration, and quality control samples

Primary stock solutions were obtained by dissolving the powders in different solutions, depending on their solubility. CEF, TAZ, POS, VOR, and LIN were dissolved in DMSO; SUL, TEI, CAS, TIG, and VAN were dissolved in water; PIP, MER, DAP, and FLU were dissolved in methanol. A stock solution of 10 mg/mL was prepared for each compound. Following this, 10 μL of the stock solution of each compound was transferred to a new tube, and 860 μL of blank serum was added to prepare a 100 μg/mL mixed stock solution. The samples were serially diluted with human serum to obtain concentrations of 0.1, 0.2, 0.3, 0.5, 1, 2, 5, 10, 20, 50, and 100 μg/mL according to their calibration curve. All four IS concentrations were 1 mg/mL. They were stored at −80°C until use.

Six concentration points were selected to construct the standard curve. Quality control (QC) samples of different concentrations were obtained by spiking human serum samples with an appropriate amount of the mixed stock solutions. The concentrations of standard curves and QCs were shown in Supplementary Table S2. The standards and QC solutions were divided into aliquots and stored in polypropylene tubes at −80°C until use.

### 2.6 Method validation

#### 2.6.1 Linearity

Calibration curves were constructed by plotting the peak area ratios of the six analyte concentrations and the corresponding ISs as a function of the concentration. The lower limit of quantification (LLOQ) is the minimum analyte concentration at which the signal-to-noise (S/N) ratio is greater than five, with the coefficient of variation (CV) less than 20% and accuracy between 80% and 120%, respectively.

#### 2.6.2 Accuracy and precision

The accuracy and precision were assessed at four levels of analyses (*n* = 6) for the QC samples and measured on three separate days. The CV, which measures the accuracy and precision within and between runs, should be less than 15% for low-quality control (LQC), medium-quality control (MQC), and high-quality control (HQC)), and less than 20% for LLOQ. (Food and Drug Administration, 2018).

#### 2.6.3 Selectivity

The selectivity was determined by examining blank serum samples collected from six healthy volunteers; these serum samples did not contain the tested antibacterial agents. Besides, each of the two hemolytic/icteric/lipemic (HIL) serum specimens from critically ill patients (who did not receive the analytes of interest) was also collected and pooled and used as blank serum. The selectivity was assessed by injecting ISs into blank samples and monitoring the signals of the target analytes in the channel.

#### 2.6.4 Matrix effect and extraction recovery

As stated in the guidelines, six biological serum matrices from various sources were spiked with analytes and ISs after extraction to assess the matrix effects. The protocol established by Matuszewski et al. was followed for this process. (Matuszewski et al., 2003). The pooled HIL blank serum specimens were also tested. The IS normalized matrix effect was calculated as the ratio of the peak areas of the analytes spiked after extraction against those of pure solutions with the same concentration, which must be less than 20% for the relative standard deviation of the normalized factors.

Extraction recoveries were evaluated by comparing the peak areas of the analytes spiked in blank serum before and after extraction at the LQC and HQC levels. The extraction recoveries were determined from replicate analyses (*n = 3*).

#### 2.6.5 Stability

Stability was widely studied under different situations. To determine the ideal conditions for the preanalytical phase, the short-term stability was examined in triplicate for the spiked serum samples. Briefly, the signals of samples after storage in the refrigerator (4°C) for 48 h were compared with that of freshly prepared QCs. The freeze−thaw stability was evaluated by subjecting the HQC and LQC samples to four freeze–thaw cycles for at least 6 h at room temperature and 12 h at −80°C. The long-term stability of all the antibacterial agents tested was evaluated for the spiked serum samples stored at −80°C for 3 months. According to Food and Drug Administration (FDA) standards, less than 15% changes in concentration are considered acceptable stabilities. (International Council for Harmonization, 2019).

### 2.7 Statistical analysis

Data analysis was performed using Microsoft Excel 2016. Statistical analyses were performed using the SPSS 21.0 software.

## 3 Results and discussion

### 3.1 Method development

**FIGURE 1:**
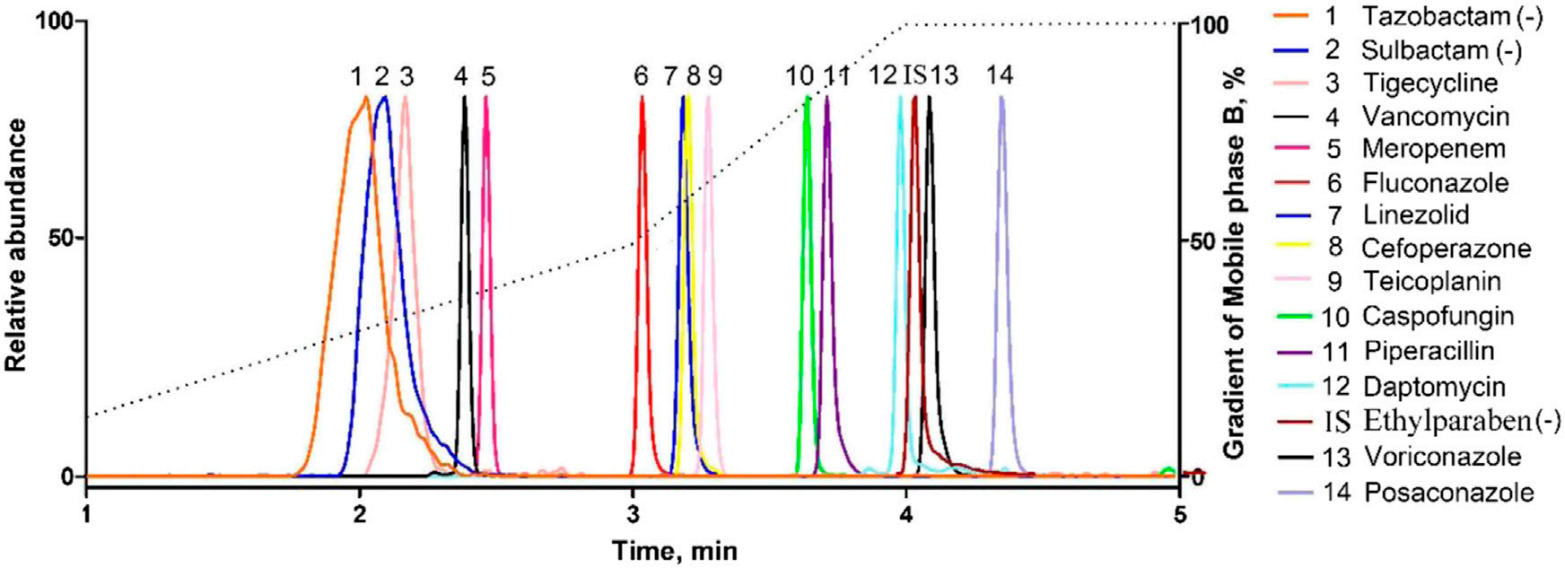
Representative MRM chromatograms obtained from human serum samples spiked with antibacterial agents. All compounds were well separated within 5 min.

All the parent ions and product ions are listed in Table 1. Every analyte exhibits two transitions, one for quantitation, and the other for confirmation. In the positive ionization mode, the intensities of the protonated molecular peaks of all compounds were higher than the peak intensities of the corresponding molecular ions produced by deprotonation in the negative ionization mode, except for SUL, TAZ. Ideally, the molecular weight of sodium salt and crystalline water should be subtracted from the molecular weight of their parent ion. For example, these can include the sodium salt of CEF and SUL and crystalline water from PIP. Three different columns (Agilent SB C_18_: 2.7 μm, 2.1 mm × 30 mm; Waters Acquity UPLC C_8_: 1.7 μm, 2.1 mm × 50 mm; and SHIMADZU C_8:_ 2 μm, 2.1 mm × 100 mm) were tested, and the column affording the optimal separation was adopted. The Waters Acquity UPLC C_8_ column was found to be superior for retaining aqueous compounds*,* with better chromatographic separation. The mobile phases initially used were water with 0.1% FA and acetonitrile with 0.1% FA (Phase B). Different concentrations of ammonium acetate (2 and 10 mM) were added to the mobile phases to identify compounds that were sensitive to pH variation and to obtain better peak shapes. However, no remarkable improvement was observed compared to the peaks obtained using the mobile phase without ammonium derivatives. In the initial stages of this study, the retention times of MER and VAN using the first gradient tested (initial mobile phase B concentration 10%; held constant for 1 min; increased to 90% in 4 min; held constant for 4 min; decreased to 10% in 1 min) were very short. After examining different gradients, the gradient was finally set as follows: 5% (0 min) −50% (3 min)–100% (4 min)–100% (7 min)– 5% (9 min)–5% (10 min). Under these conditions, the compounds could be identified and quantified based on well-resolved peaks. The chromatogram is shown in Figure 1.

**TABLE 1:** Retention times, ionization conditions, linear ranges, and corresponding ISs for 14 antibacterial agents.

Compound	Retention Time (min)	Precursor (m/z)	Product (m/z) (ion ratio,%)	Collision Energy (eV)	Linear Range (μg/mL)	IS
FLU	3.09	307.1	220.1*	−19	0.1–50	DAT-IS
			238.1 (96.9)	−16		
LIN	3.24	338.1	296.1*	−18	0.1–50	VOR-IS
			148.1 (52.7)	−43		
CAS	4.65	547.4	137.1*	−30	0.1–100	VOR-IS
			131.1 (83.0)	−26		
MER	2.53	384.1	141.1*	−16	0.1–50	VOR-IS
			114.0 (68.5)	−36		
TIG	2.23	586.3	513.2*	−27	0.1–100	VOR-IS
			456.15 (42.1)	−35		
PIP	3.73	518.2	143.2*	−52	0.1–50	PI-IS
			115.1 (25.9)	−52		
CEF	3.21	646.5	143.0*	−50	0.1–100	VOR-IS
			148.1 (28.0)	−50		
TEI	3.35	940.5	316.4*	−41	0.3–100	DAT-IS
			144.1 (56.2)	−41		
POS	4.41	701.3	614.3*	−36	0.1–50	VOR-IS
			344.2 (54.6)	−47		
VOR	4.12	350.2	281.2*	−12	0.1–50	VOR-IS
			127.2 (70.3)	−12		
DAP	4.02	810.7	159.1*	−49	0.3–100	DAT-IS
			341.1 (49.0)	−24		
VAN	2.42	725.6	144.2*	−22	0.1–100	DAT-IS
			100.1 (12.2)	−33		
SUL	2.01	232.1	140.1*	13	0.1–50	ETH
			64.0 (41.0)	35		
TAZ	2.12	299.1	137.9*	14	0.1–50	ETH
			254.9 (71.7)	10		
PI-IS	3.73	522.7	148.2*	−10	—	—
			160.2 (17.4)	−20		
VOR-IS	4.12	355.3	128.2*	−20	—	—
			284.1 (47.9)	−35		
DAT-IS	4.02	813.1	163.2*	−35	—	—
			317.2 (45.9)	−35		
ETH	4.15	165.15	92.00*	35	—	—
			136.75 (22)	20		

FLU, fluconazole; LIN, linezolid; CAS, caspofungin; MER, meropenem; TIG, tigecycline; PIP, piperacillin; CEF, cefoperazone; TEI, teicoplanin; POS, posaconazole; VOR, voriconazole; DAP, daptomycin; VAN, vancomycin; SUL, sulbactam; TAZ, tazobactam; VOR-IS, [^13^C_2_,^2^H_3_]-voriconazole; PIP-IS, [^2^H_5_]-piperacillin sodium salt; DAP-IS, daptomycin-d_5_, trifluoroacetic; ETH, ethylparaben. Product ion: the product ion designated by* for each analyte was utilized for quantification while the other was used for confirmation. Ion ratio: the relative abundance of confirmation ion intensity to quantitation ion intensity at 1 μg/mL.

Subsequently, six different organic solvents (methanol, methanol/acetonitrile (50:50), acetonitrile, methanol (0.1% FA), methanol/acetonitrile (50:50, 0.1% FA), and acetonitrile (0.1% FA) were used for protein precipitation to optimize sample pretreatment. The extraction recovery of all the analytes was calculated as the sample response ratio of the drug added before protein precipitation to that added after protein precipitation in the same solution. Pretreatment with methanol containing 0.1% FA was selected, as a superior recovery value and consistent results for all the compounds could be achieved compared to that using other solvents.

The matrix effect is important because it may affect the quantification precision, particularly with electrospray ionization (ESI). When possible, the utilization of 14 isotope-labeled ISs is an ideal choice. Generally, the selection of an IS is based on its ability to correct and reproduce the analytical behavior of each antibacterial agent. In this study, PIP-D5, DAP-D5, VOR-D5, and ETH were chosen as the ISs to balance cost and accuracy. Furthermore, we found that these four ISs were stably detected in all measurements and exhibited good performance for standard curve correction.

### 3.2 Method validation

#### 3.2.1 Linearity, precision, and accuracy

**FIGURE 2:**
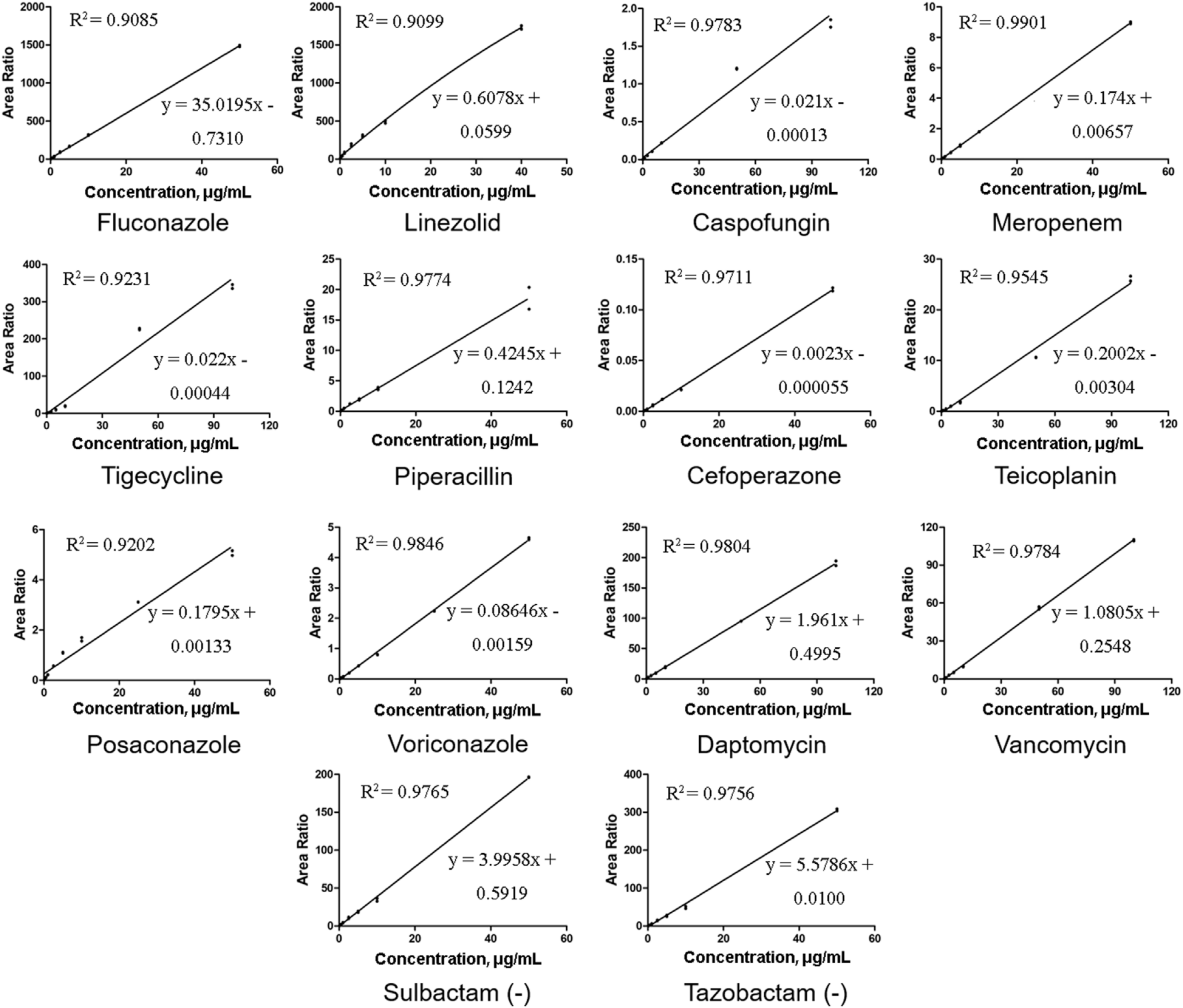
Representative calibration curves of 14 antibacterial agents. All the correlation coefficient (*R* ^2^) values were at least 0.9085 or higher.

The relative peak ratios of the analyte and ISs were plotted against the analyte concentrations, and calibration curves were generated using least-squares regression; linear regression with a weighting factor of *1*/*x* ^*2*^ was employed. (Gu et al., 2014). In our study, there was a good linear correlation, and the correlation coefficients (*R* ^2^) of all the calibration curves were 0.9,085 or higher. Representative calibration curves are shown in Figure 2. The ranges of LIN, POS, VOR, and other compounds were within a limited concentration (50 μg/mL) to avoid signal saturation at higher concentrations or exceeding the clinically effective concentration range. According to the bioanalytical guidelines of FDA, the analytical signal corresponding to the LOQ was at least five times greater than that of the above blank matrix at its retention time window. The LLOQ in our study was higher than the FDA-defined LOQ because the routinely used TDM doesn’t require a highly sensitive method well below the effective concentration range. Therefore, we adjusted our concentration range, although we were able to determine lower concentrations. The LLOQ of our method was 0.1 μg/mL for most molecules, and 0.3 μg/mL only for DAP and TEI.

The accuracy and precision of the 14 antibacterial assays were examined by four levels of analyses (*n = 6*) of the QC serum samples. Table 2 summarizes the intra- and inter-day precisions for all the drugs. For LLOQ, the intra-day precision ranged from 5.83% to 12.96%, inter-day precision ranged from 8.43% to 14.78%, and mean bias values ranged from −10.06% to 13.70%; all the concentrations satisfied the FDA recommendations for LLOQs. For the other QC samples, the intra-day precision ranged from 2.57% to 11.82%, inter-day precision ranged from 3.36% to 14.93%, and the mean bias values ranged from 2.62% to 12.73%; all the values were within the FDA recommendations, —i.e., ±15% for precision and accuracy.

**TABLE 2:** Accuracy and precision of the developed method.

Compound	LLOQ					LQC					MQC					HQC				
	C (μg/mL)	Mean (μg/mL)	% Bias	Intra-day Precision (%CV)	Inter-day Precision (%CV)	C (μg/mL)	Mean (μg/mL)	% Bias	Intra-day Precision (%CV)	Inter-day Precision (%CV)	C (μg/mL)	Mean (μg/mL)	% Bias	Intra-day Precision (%CV)	Inter-day Precision (%CV)	C (μg/mL)	Mean (μg/mL)	% Bias	Intra-day Precision (%CV)	Inter-day Precision (%CV)
FLU	0.10	0.1052	5.20	9.26	8.78	0.30	0.3219	7.32	6.98	10.29	5.00	5.475	9.52	9.47	12.21	40.00	43.87	9.68	7.31	11.17
LIN	0.10	0.1137	13.70	11.92	13.19	0.30	0.3029	0.97	8.93	5.37	5.00	4.682	−6.36	6.82	7.92	40.00	42.27	5.68	3.82	2.62
CAS	0.10	0.0936	−6.40	9.78	14.26	0.30	0.2749	−8.37	4.01	7.38	10.00	10.25	2.52	11.35	12.73	80.00	78.21	−2.24	3.34	6.26
MER	0.10	0.1046	4.61	6.72	8.43	0.30	0.2926	−2.47	8.29	11.29	5.00	4.917	−1.66	7.89	11.95	40.00	39.47	−1.33	2.57	8.10
TIG	0.10	0.0987	−1.30	9.74	12.15	0.30	0.3171	5.70	6.52	8.91	10.00	10.83	8.33	9.22	9.26	80.00	76.72	−1.41	8.91	9.27
PIP	0.10	0.1129	12.92	12.96	14.78	0.30	0.3182	6.07	9.24	11.68	5.00	4.793	−4.14	11.03	12.82	40.00	42.40	6.12	10.21	8.74
CEF	0.10	0.1107	10.73	11.83	13.64	0.30	0.2814	−6.21	10.21	9.27	10.00	9.68	−3.21	8.62	11.24	80.00	77.27	−3.41	5.28	12.18
TEI	0.30	0.2912	−2.93	8.82	11.92	0.90	0.9271	3.01	6.43	8.92	10.00	11.24	12.40	8.72	9.26	80.00	83.62	7.35	8.20	9.07
POS	0.10	0.1123	11.23	12.63	12.62	0.30	0.3239	7.97	9.63	10.71	5.00	4.631	−7.38	5.92	9.72	40.00	44.73	11.82	5.32	8.23
VOR	0.10	0.1086	8.61	7.93	8.34	0.30	0.3226	7.53	3.54	7.63	5.00	5.538	10.76	11.82	6.92	40.00	42.81	7.03	4.68	6.28
DAP	0.30	0.3314	10.47	9.81	14.29	0.90	0.8980	−0.22	8.72	9.46	10.00	11.29	12.93	6.83	11.36	80.00	83.49	10.68	8.72	11.45
VAN	0.10	0.1118	11.80	5.83	9.29	0.30	0.2824	−5.87	5.29	10.82	10.00	8.83	−11.68	9.72	11.25	80.00	82.36	2.95	9.03	8.20
SUL	0.10	0.1048	4.82	8.79	11.03	0.30	0.3161	5.37	9.83	9.31	5.00	4.871	−2.58	6.72	9.07	40.00	42.48	6.22	8.52	9.25
TAZ	0.10	0.0894	−10.06	12.66	10.72	0.30	0.3107	3.57	6.10	8.94	5.00	5.234	4.68	5.89	11.26	40.00	37.28	−6.83	11.45	10.26

#### 3.2.2 Specificity and selectivity

**FIGURE 3:**
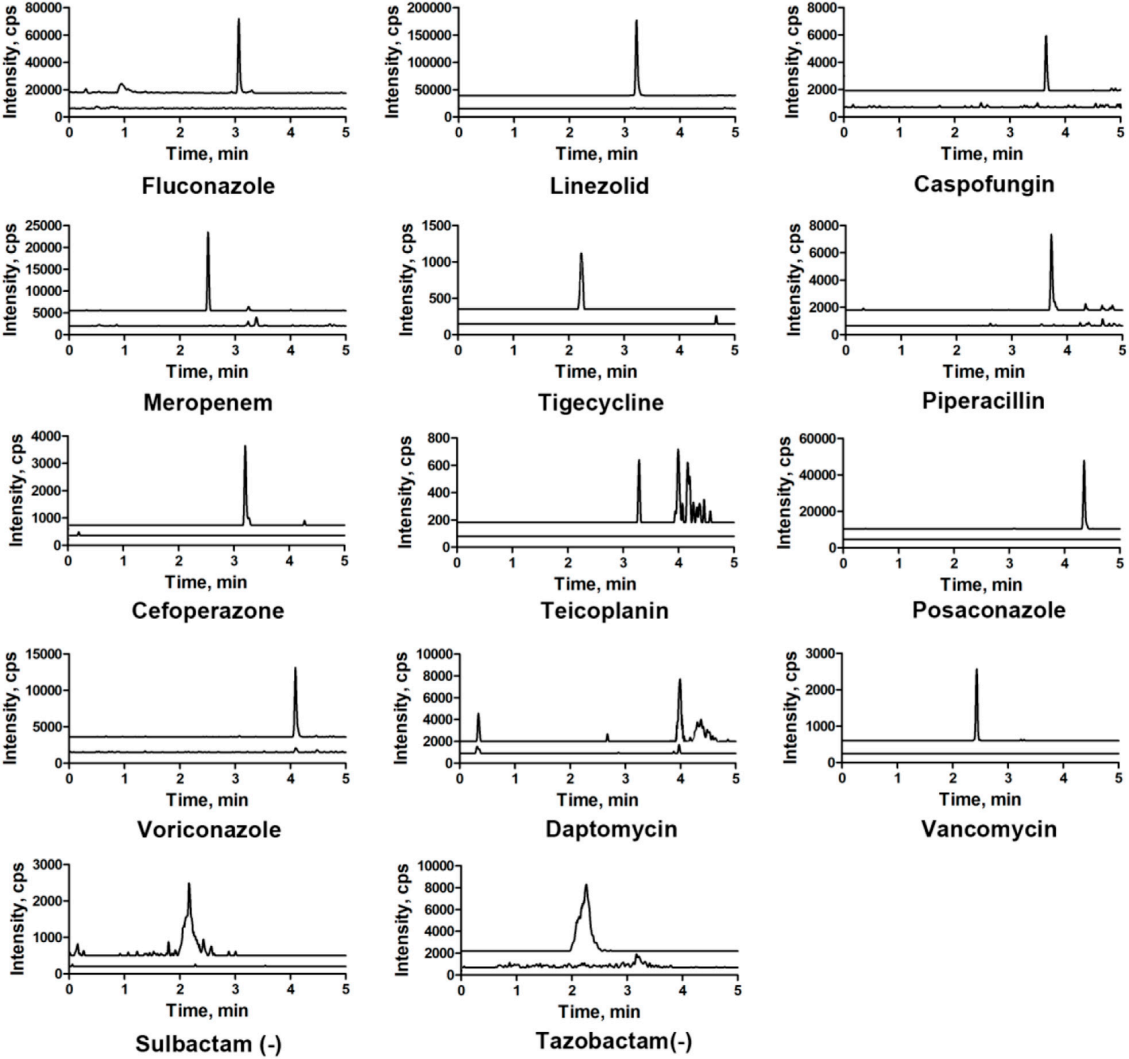
Overlapping of the LLOQ and blank-extracted chromatograms of 14 antibacterial agents.

Chromatograms of typical blank samples and LLOQ samples for all the compounds are shown in Figure 3. In both, the healthy and HIL serum spiked samples, no endogenous interferences were observed in any of the batches screened in the retention time windows for each specified MRM.

#### 3.2.3 Extraction recovery and matrix effect

The mean extraction recoveries calculated for low and high QC concentrations (three-fold LLOQ and 80% ULOQ) are summarized in Table 3. The data show that the recoveries of most compounds are greater than 80%; only LIN, CEF, and SUL show lower recoveries of 61.61%, 69.25%, and 49.42%, respectively. Additionally, the relative standard deviations (RSDs) of the recoveries are within 15%. The HIL serum spiked samples were also tested, and no difference was observed. The matrix effect in ESI-MS is an essential factor that must be considered, especially when quantifying a large number of compounds in the same bioanalytical run. In our study, the matrix factors ranged from 0.46 to 2.24 at low and high QC concentrations. The mean matrix effect for these compounds was reproducible and less than 15% for all the tested analytes.

**TABLE 3:** Extraction recovery and matrix effect of the developed method.

Compound	Recovery%				Matrix effect			
	LQC	RSD	HQC	RSD	LQC	RSD	HQC	RSD
FLU	92.37	12.30	86.31	4.32	1.33	10.91	1.59	4.29
LIN	74.23	13.45	61.61	2.71	1.77	9.38	1.52	5.77
CAS	91.02	11.74	88.23	11.72	2.24	12.50	0.46	9.98
MER	87.12	10.24	85.47	4.92	1.24	10.00	1.27	4.42
TIG	84.64	12.19	79.22	6.28	0.81	12.33	0.79	3.95
PIP	104.27	9.43	98.57	12.28	0.46	12.97	0.73	9.92
CEF	69.25	11.35	79.83	5.38	1.62	12.21	1.54	4.48
TEI	114.23	8.74	121.66	4.97	0.76	9.87	0.84	3.32
POS	87.65	9.33	79.97	2.16	1.21	8.42	1.32	2.46
VOR	93.63	10.65	115.24	4.13	1.58	4.99	1.47	4.30
DAP	84.83	9.74	76.43	5.86	0.67	13.94	0.50	13.54
VAN	108.60	10.45	101.63	6.87	1.30	9.41	1.61	4.14
SUL	58.74	12.09	49.42	1.75	1.43	8.08	1.56	9.84
TAZ	110.81	9.38	93.57	3.88	1.56	14.65	1.52	4.12

RSD, relative standard deviation.

#### 3.2.4 Stability

The LQCs and HQCs were tested to evaluate the stability of the samples under different temperature and other conditions, and the mean and RSD of the standard solution are listed in Supplementary Table S3. (n = 6). These changes were acceptable.

### 3.3 Application

The validated method was used to examine 255 serum samples for TDM, and samples were obtained from 139 ICU patients treated with antibacterial agents at the Zhongnan Hospital of Wuhan University. The patients received the drug intravenously (except for POS, which was administered orally), and serum samples were collected just before the next administration post five to seven administrations (i.e., once the steady-state concentrations were achieved). The results are listed in Table 4 **.** The determined drug concentrations were correlated with the minimum inhibitory concentrations for optimizing the treatment in each patient according to the specific PK/PD indexes. For example, the mean value of VAN was 20.57 μg/mL, which is above the recommended range (10–20 μg/mL). This is probably because of the limited renal function in most ICU patients. These results support the need for the TDM of antibacterial agents to promote their appropriate administration.

**TABLE 4:** Results of the measurement of antibacterial agents in samples collected from ICU patients.

Compound	Samples	Patients	Mean Concentration (μg/mL, range)
FLU	12	7	11.5 (5.6–29.2)
LIN	9	5	8.3 (4.4–13.3)
CAS	11	5	5.9 (1.9–7.4)
MER	12	5	16.8 (1.3–47.2)
TIG	5	3	7.2 (4.4–10.2)
PIP	16	9	17.9 (4.8–89.9)
CEF	14	6	153.2 (68.3–220.1)
TEI	12	5	43.3 (10.3–124.5)
POS	11	6	1.4 (0.2–4.2)
VOR	13	8	1.2 (0.1–7.2)
DAP	37	21	28.37 (6.3–88.34)
VAN	67	38	20.57 (3.25–94.74)
SUL	19	12	26.38 (13.47–60.42)
TAZ	17	9	11.9 (4.1–26.9)

## 4 Conclusion

In this study, a robust, and sensitive LC–MS/MS assay was developed for the simultaneous quantification of 14 antibacterial agents in human serum. Validation indicated that the developed method exhibited good linearity and acceptable precision and accuracy. This method required as low as 100 μL of serum samples with rapid protein precipitation, and could be successfully employed for TDM of 14 antibacterial agents in ICU patients.

## Data Availability

The raw data supporting the conclusions of this article will be made available by the authors, without undue reservation.
