# Supplementary material for: LC–MS/MS-based multiplex antibacterial platform for therapeutic drug monitoring in intensive care unit patients
Source: Front Pharmacol. 2023 Apr 18;14:1116071. doi: 10.3389/fphar.2023.1116071 (PMC10151781; doi:10.3389/fphar.2023.1116071)
Supplement: Supplementary file 1 [file DataSheet1.PDF]

Supporting information

LC–MS/MS-based multiplex antibacterial platform for therapeutic drug monitoring in intensive care unit patients

*Liang Liu [1], Liu Zhang[1], Xiangyi Zheng[1], Xing Liu[2], Wei Liu[3], Jianhua Wu [1]**

[1] Department of Pharmacy, Zhongnan Hospital of Wuhan University, Wuhan, 430071, China

[2] Department of Critical Care Medicine, Zhongnan Hospital of Wuhan University, Wuhan, 430071, China

[3] School of Physics and Technology, Wuhan University, Wuhan, 430072, China

*Correspondence: Wu Jianhua, Department of Pharmacy, Zhongnan Hospital of Wuhan University, 169 Donghu Road, Wuchang District, Wuhan, 430071, China

Phone: 86-27-67812902

Email: jhwu@whu.edu.cn

Table of Contents:

Table S1. Gradient of mobile phase.

Table S2. Concentrations for calibration curves and QCs (μg/mL).

Table S3. Stability of QC samples under different conditions, data are represented as mean (percentage).

Table 1S. Gradient of mobile phase.

Time (min)	Mobile Phase B (%)	Mobile Phase A (%)
0	5	95
0.5	5	95
3	10	90
4	50	50
8	100	0
9	5	95
10	5	95

Table 2S. Concentrations for calibration curves and QCs (μg/mL).

Compound	level 1	level 2	level 3	level 4	level 5	level 6	LLOQ	LQC	MQC	HQC
**FLU**	0.1	0.2	0.5	2	10	50	0.1	0.3	5	40
**LIN**	0.1	0.2	0.5	2	10	50	0.1	0.3	5	40
**CAS**	0.1	0.2	1	4	20	100	0.1	0.3	10	80
**MER**	0.1	0.2	0.5	2	10	50	0.1	0.3	5	40
**TIG**	0.1	0.2	1	4	20	100	0.1	0.3	10	80
**PIP**	0.1	0.2	0.5	2	10	50	0.1	0.3	5	40
**CEF**	0.1	0.2	1	4	20	100	0.1	0.3	10	80
**TEI**	0.3	0.5	1	4	20	100	0.3	0.9	10	80
**POS**	0.1	0.2	0.5	2	10	50	0.1	0.3	5	40
**VOR**	0.1	0.2	0.5	2	10	50	0.1	0.3	5	40
**DAP**	0.3	0.5	1	4	20	100	0.3	0.9	10	80
**VAN**	0.1	0.2	1	4	20	100	0.1	0.3	10	80
**SUL**	0.1	0.2	0.5	2	10	50	0.1	0.3	5	40
**TAZ**	0.1	0.2	0.5	2	10	50	0.1	0.3	5	40

Table 3S. Stability of QC samples under different conditions, data are represented as mean (percentage).

Compound	Concentration (μg/mL)		Freshly prepared QC (μg/mL)		4°C for 48 h (μg/mL)		Four freeze-thraw cycles (μg/mL)		-80°C for 3 months (μg/mL)	
	LQC	HQC	LQC	HQC	LQC	HQC	LQC	HQC	LQC	HQC
**FLU**	0.30	40.00	0.31 (104.41)	42.57 (106.43)	0.28 (93.87)	40.22 (100.55)	0.28 (94.67)	39.63 (99.08)	0.31 (104.67)	40.81 (102.03)
**LIN**	0.30	40.00	0.30 (101.27)	37.98 (94.95)	0.27 (90.48)	38.77 (96.93)	0.28 (93.67)	36.83 (92.08)	0.29 (97.00)	37.25 (93.13)
**CAS**	0.30	80.00	0.32 (107.83)	81.47 (101.84)	0.29 (96.67)	78.48 (98.10)	0.29 (97.00)	76.54 (95.68)	0.28 (92.67)	83.36 (104.2)
**MER**	0.30	40.00	0.29 (96.14)	39.98 (99.95)	0.27 (91.33)	37.34 (93.35)	0.27 (91.23)	35.23 (88.08)	0.27 (91.00)	36.24 (90.60)
**TIG**	0.30	80.00	0.33 (109.25)	85.58 (106.98)	0.29 (97.24)	76.49 (95.61)	0.29 (97.23)	80.44 (100.55)	0.30 (100.33)	78.99 (98.74)
**PIP**	0.30	40.00	0.28 (94.16)	39.77 (99.43)	0.31 (103.79)	39.54 (98.85)	0.30 (99.28)	37.39 (93.48)	0.31 (104.17)	36.74 (91.85)
**CEF**	0.30	80.00	0.30 (99.23)	74.47 (93.09)	0.30 (99.53)	72.39 (95.83)	0.31 (103.33)	73.68 (92.10)	0.29 (97.97)	79.32 (99.15)
**TEI**	0.90	80.00	0.92 (103.24)	83.44 (104.30)	0.83 (92.22)	81.75 (102.19)	0.84 (92.00)	75.88 (94.85)	0.83 (92.33)	76.39 (95.49)
**POS**	0.30	40.00	0.29 (95.37)	38.24 (95.60)	0.31 (104.11)	38.93 (97.33)	0.31 (104.23)	39.03 (97.58)	0.30 (101.63)	38.61 (96.53)
**VOR**	0.30	40.00	0.31 (103.23)	42.87 (107.18)	0.29 (97.00)	40.93 (102.33)	0.28 (92.67)	39.47 (98.68)	0.28 (94.85)	38.55 (96.38)
**DAP**	0.90	80.00	0.89 (98.89)	82.42 (103.03)	0.88 (97.78)	78.44 (98.05)	0.85 (94.67)	80.46 (100.58)	0.82 (91.10)	78.26 (97.83)
**VAN**	0.30	80.00	0.29 (96.67)	78.31 (97.89)	0.29 (95.98)	76.28 (95.35)	0.31 (103.67)	77.98 (97.48)	0.32 (107.00)	76.38 (95.48)
**SUL**	0.30	40.00	0.30 (101.22)	40.32 (100.80)	0.30 (98.95)	39.88 (99.70)	0.28 (94.00)	38.24 (95.60)	0.33 (108.93)	37.25 (93.13)
**TAZ**	0.30	40.00	0.29 (96.34)	38.75 (96.88)	0.31 (103.45)	38.23 (95.58)	0.27 (91.33)	37.77 (94.43)	0.28 (93.84)	38.47 (96.18)
